# *In Vitro* Activity of Sulbactam-Durlobactam against Global Isolates of *Acinetobacter baumannii*-*calcoaceticus* Complex Collected from 2016 to 2021

**DOI:** 10.1128/aac.00781-22

**Published:** 2022-08-25

**Authors:** James A. Karlowsky, Meredith A. Hackel, Sarah M. McLeod, Alita A. Miller

**Affiliations:** a IHMA, Inc., Schaumburg, Illinois, USA; b Department of Medical Microbiology and Infectious Diseases, Max Rady College of Medicine, University of Manitoba, Winnipeg, Manitoba, Canada; c Entasis Therapeutics, Waltham, Massachusetts, USA

**Keywords:** sulbactam-durlobactam, ETX2514, *Acinetobacter baumannii-calcoaceticus* complex, carbapenem-resistant, multidrug-resistant

## Abstract

Sulbactam-durlobactam is a β-lactam-β-lactamase inhibitor combination designed to treat serious Acinetobacter baumannii*-calcoaceticus* complex (ABC) infections, including carbapenem-non-susceptible and multidrug-resistant (MDR) isolates. The current study characterized the *in vitro* activity of sulbactam-durlobactam against a collection of 5,032 ABC clinical isolates collected in 33 countries across the Asia/South Pacific region, Europe, Latin America, the Middle East, and North America from 2016 to 2021. The sulbactam-durlobactam MIC_50_ and MIC_90_ were 1 and 2 μg/mL, respectively, for all ABC isolates tested. The addition of durlobactam (at a fixed concentration of 4 μg/mL) to sulbactam decreased its MIC_50_ by 8-fold (from 8 to 1 μg/mL) and its MIC_90_ by 32-fold (from 64 to 2 μg/mL) for all ABC isolates. The *in vitro* activity of sulbactam-durlobactam was maintained across individual ABC species, years, global regions of collection, specimen sources, and resistance phenotypes, including MDR and extensively drug-resistant (XDR) isolates. At 4 μg/mL (preliminary sulbactam-durlobactam susceptible MIC breakpoint), sulbactam-durlobactam inhibited 98.3% of all ABC isolates and >96% of sulbactam-, imipenem-, ciprofloxacin-, amikacin-, and minocycline-non-susceptible isolates; as well as colistin-resistant, MDR, and XDR isolates. Most imipenem-non-susceptible ABC isolates (96.8%, 2,488/2,570) were carbapenem-resistant A. baumannii (CRAB); 96.9% (2,410/2,488) of CRAB isolates were sulbactam-durlobactam-susceptible. More than 80% of ABC isolates had sulbactam-durlobactam MIC values that were ≥2 doubling-dilutions (4-fold) lower than sulbactam alone. Only 1.7% (84/5,032) of ABC isolates from 2016 to 2021 had sulbactam-durlobactam MIC values of >4 μg/mL. Of the 84 isolates, 94.0% were A. baumannii, 4.8% were A. pittii, and 1.2% were A. nosocomialis. In summary, sulbactam-durlobactam demonstrated potent antibacterial activity against a 2016 to 2021 collection of geographically diverse clinical isolates of ABC isolates, including carbapenem-non-susceptible and MDR isolates.

## INTRODUCTION

Acinetobacter baumannii*-calcoaceticus* complex (ABC) species (A. baumannii, A. calcoaceticus, A. dijkshoorniae, A. nosocomialis, A. pittii, A. seifertii) are the most medically important phylogroup of the genus Acinetobacter (1, 2). The majority of ABC clinical isolates are A. baumannii. ABC species are well-documented pathogens in nosocomial outbreaks and cause severe infections with high mortality rates that are often linked to aspiration and ventilator-associated pneumonia and catheter-associated bacteremia following intensive care unit admission (1–3). Globally, the susceptibility of ABC species to all first- and second-line antimicrobial agents used to treat Acinetobacter infections has declined over the last 30 years with many isolates (primarily A. baumannii) now testing as multidrug-resistant (MDR) or extensively drug-resistant (XDR) (4, 5). The identification and development of new agents to treat antimicrobial-resistant ABC infections, for which there is a high unmet medical need, is an international priority (6).

Sulbactam-durlobactam (formerly sulbactam-ETX2514) is a narrow-spectrum, parenteral β-lactam-β-lactamase inhibitor combination that recently completed a phase 3 study to evaluate its safety and efficacy for the treatment of serious infections caused by ABC, including carbapenem-resistant and MDR isolates (7, 8). Sulbactam-durlobactam has been designated a Qualified Infectious Disease Product (QIDP) by the United States Food and Drug Administration and awarded Fast Track status.

Sulbactam, a semi-synthetic penicillanic acid, is a β-lactamase inhibitor of a subset of Ambler class A enzymes (excluding TEM-1). It was initially partnered with ampicillin in the 1980s and was approved for skin and skin structure, intra-abdominal, bone and joint, and gynecological infections. Sulbactam also inhibits bacterial cell wall synthesis in ABC by binding to penicillin-binding protein (PBP) 1a/b and PBP3 (9). Sulbactam is susceptible to degradation by a variety of acquired or upregulated β-lactamases, including the serine β-lactamases of class A (TEM-1), class C (ADC-30), and class D (OXA), and class B metallo-β-lactamases (MBLs) (8). Resistance to sulbactam as well as broad-spectrum cephalosporins and carbapenems has emerged in ABC and spread widely, mainly due to the acquisition of OXA β-lactamases (OXA-23, OXA-24/40, OXA-51, OXA-58, OXA-143, OXA-235) (1, 2, 5).

Durlobactam is a rationally designed non-β-lactam diazabicyclooctane (DBO) β-lactamase inhibitor of Ambler class A, C, and D β-lactamases that can protect sulbactam from degradation by these enzymes, effectively restoring its activity against sulbactam-non-susceptible ABC isolates expressing these β-lactamases (10–16). Durlobactam does not inhibit MBLs (10). Durlobactam has a modified DBO scaffold resulting in inhibition of a broad range of class D β-lactamases, with notably more potent inhibition of class A and C β-lactamases compared to other DBO inhibitors (e.g., avibactam, relebactam) (10, 11). Ceftazidime-avibactam, imipenem-relebactam, meropenem-vaborbactam, and ceftolozane-tazobactam do not have clinically useful activity against Acinetobacter spp. (17).

The goal of the current study was to characterize the *in vitro* activity of sulbactam-durlobactam against a recent geographically diverse collection of clinical ABC isolates. Study isolates were chosen from −70°C frozen stocks maintained by International Health Management Associates (IHMA; Schaumburg, IL) based on geographic distribution, site of infection, and year of isolation (and therefore, this was not designed to be a prevalence-based study).

## RESULTS

The current study surveyed 5,032 ABC isolates collected by clinical laboratories in 264 medical centers in 33 countries across five global regions (Europe, 42.2% of all isolates tested; North America [United States], 29.9%; Asia/South Pacific, 13.6%; Latin America, 12.6%; Middle East [Israel], 1.7%) from 2016 to 2021 and determined their *in vitro* susceptibility to sulbactam-durlobactam and nine comparator agents. The percentages of all isolates tested by year were 16.8% from 2016, 16.4% from 2017, 18.4% from 2018, 17.1% from 2019, 15.8% from 2020, and 15.5% from 2021. Isolates tested were limited to one isolate per patient and were primarily from five common infection sources: lower respiratory (54.3% of all isolates tested), bloodstream (20.2%), urinary tract (16.5%), skin and soft tissue (4.5%), and intraabdominal (4.3%). To be consistent with clinical experience, (1–3) 80.2% of the isolates in the survey were A. baumannii, followed by 12.7% A. pittii, 5.9% A. nosocomialis, and 1.1% A. calcoaceticus.

The sulbactam-durlobactam MIC_50_ and MIC_90_ were 1 and 2 μg/mL, respectively, for all ABC isolates tested (Table 1). The addition of durlobactam (at a fixed concentration of 4 μg/mL) to sulbactam decreased its MIC_50_ by 8-fold (from 8 to 1 μg/mL) and its MIC_90_ by 32-fold (from 64 to 2 μg/mL) for all ABC isolates. MIC_50_ and MIC_90_ values for sulbactam-durlobactam ranged from 0.5 to 1 μg/mL and from 1 to 2 μg/mL, respectively, for individual ABC species. The sulbactam-durlobactam MIC range was widest for A. baumannii (≤0.03 to >64 μg/mL), narrower for A. pittii (≤0.03 to 32 μg/mL) and A. nosocomialis (≤0.03 to 8 μg/mL), and narrowest for A. calcoaceticus (0.12 to 2 μg/mL), which correlated with the number of isolates tested for each species.

**TABLE 1:** *In vitro* activities of sulbactam-durlobactam and comparator antimicrobial agents tested against 5,032 clinical isolates of Acinetobacter baumannii*-calcoaceticus* complex species collected globally from 2016 to 2021^*a*^

Species (no. of isolates)	Antimicrobial agent	MIC (μg/mL)			MIC interpretation (%)		
		MIC_50_	MIC_90_	Range	Susceptible	Intermediate	Resistant
All isolates (5,032)^*b*^	Sulbactam-durlobactam^*c*^	1	2	≤0.03–>64	98.3	NA	1.7
	Sulbactam^*d*^	8	64	0.25–>64	46.9	8.0	45.1
	Cefepime	16	>16	≤0.12–>16	44.6	7.9	47.4
	Imipenem	8	>64	≤0.03–>64	48.9	0.6	50.5
	Meropenem	16	>64	≤0.03–>64	47.9	1.1	51.0
	Amikacin	4	>64	≤0.5–>64	58.6	3.3	38.1
	Ciprofloxacin	>4	>4	≤0.12–>4	44.4	0.7	54.9
	Colistin^*e*^	0.5	1	≤0.25–>8	NA	95.9	4.1
	Minocycline	0.5	16	≤0.12–>16	78.3	10.1	11.6
	Tigecycline^*f*^	0.5	2	0.03–32	NA	NA	NA
							
A. baumannii (4,038)	Sulbactam-durlobactam	1	2	≤0.03–>64	98.0	NA	2.0
	Sulbactam	16	64	0.25–>64	36.5	8.9	54.6
	Cefepime	>16	>16	≤0.12–>16	33.6	8.8	57.6
	Imipenem	32	>64	≤0.03–>64	37.7	0.6	61.6
	Meropenem	64	>64	≤0.03–>64	36.6	1.2	62.3
	Amikacin	32	>64	≤0.5–>64	49.5	3.8	46.6
	Ciprofloxacin	>4	>4	≤0.12–>4	32.7	0.7	66.6
	Colistin	0.5	1	≤0.25–>8	NA	95.1	4.9
	Minocycline	1	16	≤0.12–>16	73.3	12.4	14.4
	Tigecycline	0.5	2	0.03–32	NA	NA	NA
							
A. calcoaceticus (55)	Sulbactam-durlobactam	0.5	1	0.12–2	100	NA	0
	Sulbactam	2	4	1–8	94.5	5.5	0
	Cefepime	4	8	1–>16	90.9	7.3	1.8
	Imipenem	0.25	0.25	0.12–1	100	0	0
	Meropenem	0.25	1	0.12–4	98.2	1.8	0
	Amikacin	1	2	≤0.5–16	100	0	0
	Ciprofloxacin	≤0.12	0.25	≤0.12–0.5	100	0	0
	Colistin	0.5	1	≤0.25–2	NA	100	0
	Minocycline	≤0.12	0.25	≤0.12–0.25	100	0	0
	Tigecycline	0.12	0.25	0.03–1	NA	NA	NA
							
A. nosocomialis (296)	Sulbactam-durlobactam	0.5	1	≤0.03–8	99.7	NA	0.3
	Sulbactam	2	16	0.25–>64	81.8	8.1	10.1
	Cefepime	2	>16	0.5–>16	85.1	4.4	10.5
	Imipenem	0.25	0.5	0.06–>64	92.2	0	7.8
	Meropenem	0.25	1	0.06–>64	92.2	0.3	7.4
	Amikacin	2	8	≤0.5–>64	92.6	2.0	5.4
	Ciprofloxacin	0.25	2	≤0.12–>4	89.9	1.4	8.8
	Colistin	0.5	1	≤0.25–>8	NA	98.0	2.0
	Minocycline	≤0.12	0.5	≤0.12–16	98.0	1.4	0.7
	Tigecycline	0.12	1	0.03–4	NA	NA	NA
							
A. pittii (638)	Sulbactam-durlobactam	0.5	2	≤0.03–32	99.4	NA	0.6
	Sulbactam	2	4	0.5–>64	92.9	2.4	4.7
	Cefepime	4	8	≤0.12–>16	91.4	4.2	4.4
	Imipenem	0.25	0.5	0.06–>64	95.1	0.3	4.5
	Meropenem	0.5	1	≤0.03–>64	95.0	0.8	4.2
	Amikacin	1	4	≤0.5–>64	96.6	1.1	2.4
	Ciprofloxacin	≤0.12	0.5	≤0.12–>4	92.8	0.3	6.9
	Colistin	0.5	1	≤0.25–4	NA	99.8	0.2
	Minocycline	≤0.12	0.25	≤0.12–16	98.9	0.6	0.5
	Tigecycline	0.12	0.5	0.03–4	NA	NA	NA

a ABC, *Acinetobacter baumannii-calcoaceticus* complex; NA, not available.

b There were four isolates of non-identified Acinetobacter spp. and one isolate of Acinetobacter dijkshoorniae that are included in the total data set but not divided out individually in the table.

c Sulbactam-durlobactam MICs were interpreted using the preliminary MIC breakpoints of ≤4 μg/mL (susceptible) and ≥8 μg/mL (resistant).

d Sulbactam MICs were interpreted using the sulbactam component of CLSI M100 (2021) ampicillin-sulbactam MIC breakpoints (≤8/4 [susceptible], 16/8 [intermediate], and ≥32/16 [resistant]) given that sulbactam is well established to comprise the active component of the combination for Acinetobacter spp.

e CLSI M100 (2021) lists only intermediate and resistant MIC breakpoints for colistin tested against Acinetobacter spp.

f MIC interpretative criteria are not published by CLSI M100 (2021) for tigecycline tested against Acinetobacter spp.

**FIG 1:**
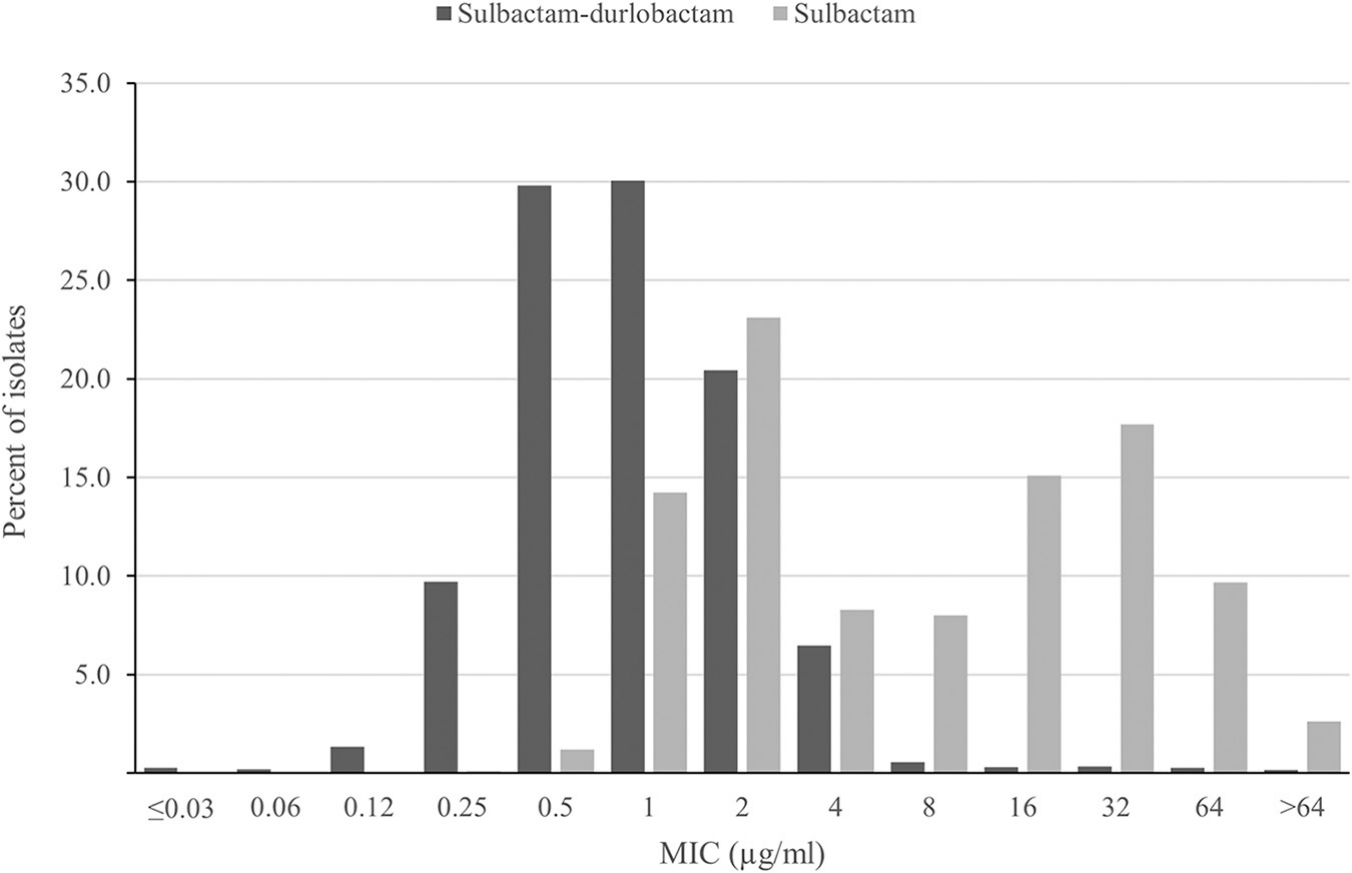
Sulbactam-durlobactam (black bars) and sulbactam (gray bars) MIC distributions for 5,032 isolates of Acinetobacter baumannii*-calcoaceticus* complex (ABC) species collected globally from 2016 to 2021.

Sulbactam-durlobactam MIC values were ≤4 μg/mL (the preliminary susceptible breakpoint) (18, 19) for 98.3% of all ABC isolates: 100% of A. calcoaceticus, 99.7% of A. nosocomialis, 99.4% of A. pittii, and 98.0% of A. baumannii isolates. Sulbactam-durlobactam demonstrated a unimodal MIC distribution, with most MICs (97.9%; 4,924/5,032) measuring from 0.12 to 4 μg/mL and with modal MICs of 0.5 and 1 μg/mL (Fig. 1). In contrast to sulbactam-durlobactam, sulbactam alone showed a bimodal MIC distribution; one population with a mode of 2 μg/mL and a range of 0.5 to 4 μg/mL and a second population with a mode of 16 to 32 μg/mL and a range of 8 to >64 μg/mL (Fig. 1). Of the 2,670 isolates with sulbactam MIC values of 8 to >64 μg/mL, 96.9% (2,587) were restored to sulbactam MIC values of ≤4 μg/mL in the presence of durlobactam, suggesting that these isolates carry β-lactamases (Table 2).

**TABLE 2:** Cumulative frequency distributions of sulbactam-durlobactam and sulbactam MICs against phenotypic subsets of antimicrobial-non-susceptible and -resistant clinical isolates of Acinetobacter baumannii*-calcoaceticus* complex collected globally from 2016 to 2021^*a*^

Antimicrobial-non-susceptible/-resistant phenotype (no. of isolates)	Cumulative percentage (%) of isolates inhibited by varying MICs (no. of isolates)^*b*^												
	MICs (μg/mL)												
	≤0.03	0.06	0.12	0.25	0.5	1	2	4	8	16	32	64	>64
All isolates (5,032)													
Sulbactam-durlobactam	0.3 (14)	0.5 (10)	1.8 (68)	11.5 (489)	41.4 (1,500)	71.4 (1,513)	**91.9 (1,029)**	98.3 (325)	98.9 (28)	99.2 (15)	99.5 (18)	99.8 (14)	100 (9)
Sulbactam				0.1 (5)	1.3 (61)	15.5 (716)	38.7 (1,163)	46.9 (417)	54.9 (403)	70.0 (759)	87.7 (890)	**97.4 (486)**	100 (132)
Sulbactam-non-susceptible^*c*^ (2,670)													
Sulbactam-durlobactam	0.1 (3)	0.1 (1)	0.4 (6)	3.0 (69)	17.0 (375)	51.7 (926)	85.2 (894)	**96.9 (313)**	97.9 (27)	98.5 (15)	99.1 (18)	99.7 (14)	100 (9)
Sulbactam									15.1 (403)	43.5 (759)	76.9 (890)	**95.1 (486)**	100 (132)
Imipenem-non-susceptible (2,570)													
Sulbactam-durlobactam	0.0 (1)	0.1 (1)	0.4 (7)	3.2 (73)	17.6 (370)	52.2 (889)	85.1 (847)	**96.7 (298)**	97.8 (28)	98.4 (15)	99.1 (18)	99.6 (14)	100 (9)
Sulbactam						0.1 (3)	0.8 (17)	3.7 (76)	16.0 (314)	43.4 (706)	76.6 (853)	**94.9 (471)**	100 (130)
Ciprofloxacin-non-susceptible (2,796)													
Sulbactam-durlobactam	0.1 (2)	0.1 (2)	0.6 (14)	3.6 (84)	18.8 (425)	53.9 (979)	86.4 (909)	**97.5 (310)**	98.3 (24)	98.8 (13)	99.2 (11)	99.7 (14)	100 (9)
Sulbactam					0.2 (5)	1.0 (23)	3.9 (82)	9.0 (143)	21.6 (350)	47.7 (732)	78.8 (867)	**95.7 (473)**	100 (121)
Amikacin-non-susceptible (2,083)													
Sulbactam-durlobactam	0.0 (1)	0.0 (0)	0.4 (7)	2.9 (53)	16.3 (279)	48.8 (676)	84.1 (735)	**96.9 (267)**	97.8 (20)	98.4 (12)	99.1 (14)	99.6 (10)	100 (9)
Sulbactam						0.2 (5)	0.6 (7)	3.9 (70)	14.2 (214)	38.8 (512)	74.0 (733)	**94.5 (427)**	100 (115)
Minocycline-non-susceptible (1,092)													
Sulbactam-durlobactam				0.6 (7)	5.1 (49)	28.7 (257)	77.5 (533)	**97.7 (221)**	98.9 (13)	99.3 (4)	99.5 (3)	100 (5)	
Sulbactam						0.1 (1)	0.3 (2)	2.8 (28)	10.6 (85)	32.6 (240)	73.3 (444)	**95.6 (244)**	100 (48)
Colistin-resistant (204)													
Sulbactam-durlobactam				2.9 (6)	11.3 (17)	44.6 (68)	79.9 (72)	**98.0 (37)**	99.5 (3)	99.5 (0)	99.5 (0)	100 (1)	
Sulbactam						2.5 (5)	5.4 (6)	8.8 (7)	20.1 (23)	43.1 (47)	72.5 (60)	**96.1 (48)**	100 (8)
MDR^*d*^ (2,680)													
Sulbactam-durlobactam	0.0 (1)	0.1 (1)	0.3 (7)	3.0 (71)	17.0 (375)	51.6 (928)	85.3 (902)	**96.9 (311)**	97.9 (28)	98.5 (15)	99.1 (18)	99.7 (14)	100 (9)
Sulbactam						0.1 (3)	0.4 (7)	4.0 (98)	17.1 (350)	44.3 (728)	77.2 (882)	**95.1 (481)**	100 (131)
XDR^*e*^ (2,116)													
Sulbactam-durlobactam			0.2 (4)	2.3 (44)	14.7 (262)	47.1 (687)	83.8 (776)	**97.2 (283)**	98.1 (20)	98.6 (11)	99.1 (10)	99.6 (10)	100 (9)
Sulbactam								0.5 (11)	11.1 (224)	37.5 (559)	74.0 (771)	**94.7 (438)**	100 (113)

a ABC, Acinetobacter baumannii*-calcoaceticus* complex; MDR, multidrug-resistant; XDR, extensively drug-resistant.

b MIC_90_ is in boldface for each MIC distribution.

c For sulbactam, a susceptibility breakpoint of ≤4 μg/mL was used, which is based on the CLSI (2021) M100 ampicillin-sulbactam (2:1) susceptible breakpoint of ≤8/4 μg/mL where sulbactam comprises the active component of the combination for Acinetobacter spp.

d MDR isolates were defined as those not susceptible to agents from ≥3 different antimicrobial classes from the following list: cefepime (extended-spectrum cephalosporins), imipenem (carbapenems), amikacin (aminoglycosides), ciprofloxacin (fluoroquinolones), minocycline (tetracycline), sulbactam (penicillin plus β-lactamase inhibitor; sulbactam is the active component of ampicillin-sulbactam against Acinetobacter spp.), and colistin (polymyxins). For colistin, only colistin-resistant isolates were used in MDR and XDR determinations because colistin-non-susceptible isolates encompass all isolates of Acinetobacter spp. (CLSI [2021] M100).

e XDR isolates were defined as those not susceptible to at least 5 of the following 7 agents or agent classes from the following list: cefepime (extended-spectrum cephalosporins), imipenem (carbapenems), amikacin (aminoglycosides), ciprofloxacin (fluoroquinolones), minocycline (tetracycline), sulbactam (penicillin plus β-lactamase inhibitor; sulbactam is the active component of ampicillin-sulbactam against Acinetobacter spp.), and colistin (polymyxins). For colistin, only colistin-resistant isolates were used in XDR determinations because colistin-non-susceptible isolates encompass all isolates of Acinetobacter spp. (CLSI [2021] M100).

Susceptibility to comparator agents varied by ABC species (Table 1). Less than 50% of A. baumannii isolates were susceptible to sulbactam, cefepime, imipenem, meropenem, amikacin, and colistin; 73.3% of isolates were minocycline-susceptible. Percentages of susceptible values were >90% for all agents tested against A. calcoaceticus and A. pittii, and for all agents except sulbactam, cefepime, and ciprofloxacin against A. nosocomialis. Colistin (MIC_90_, 1 μg/mL) and tigecycline (MIC_90_, 2 μg/mL) were the only two comparator agents tested which demonstrated *in vitro* potency equivalent to sulbactam-durlobactam against all ABC isolates tested; however, these *in vitro* potencies often do not translate into efficacy due to toxicities and suboptimal pharmacokinetics (20).

MIC_50_ and MIC_90_ values for sulbactam-durlobactam did not show appreciable differences when ABC isolates were analyzed by global region (MIC_50_ range, 1 μg/mL; MIC_90_ range, 2 to 4 μg/mL) (Table S1 in Supplemental File 1) and specimen source (MIC_50_ range, 1 μg/mL; MIC_90_ range, 2 μg/mL) (Table S2). From 2016 to 2021, MIC_90_ values for sulbactam-durlobactam for all ABC isolates fluctuated by one doubling-dilution (between 2 and 4 μg/mL) without any discernible trend (Table S3). The percentages of isolates with sulbactam-durlobactam MICs ≤4 μg/mL did not differ significantly (by <3%; *P = *0.572) across the 6 years and ranged from a low of 97.0% in 2017 to a high of 99.3% in 2018. Individual ABC species also showed random fluctuations in MIC_50_ and MIC_90_ values across years without identifiable trends.

A total of 84 ABC isolates from 2016 to 2021 had sulbactam-durlobactam MICs of >4 μg/mL (Table 2). Of these 84 isolates, 79 (94.0%) were A. baumannii, 4 (4.8%) were A. pittii, and 1 (1.2%) was A. nosocomialis. By year, the percentages of isolates with sulbactam-durlobactam MICs of >4 μg/mL were: 1.2% (10/843) in 2016, 3.0% (25/826) in 2017, 0.8% (7/928) in 2018, 2.2% (19/860) in 2019, 1.8% (14/795) in 2020, and 1.2% (9/780) in 2021. The specimen sources associated with the 84 isolates were 1.6% (16/1,015) bloodstream, 2.3% (5/217) intraabdominal, 1.9% (52/2,731) lower respiratory, 0.9% (2/227) skin and soft tissue, and 1.1% (9/832) urinary tract. The 84 isolates were spread across the five regions: 4.7% (30/632) Latin America, 2.3% (2/88) Middle East (Israel), 1.6% (11/685) Asia/South Pacific, 1.4% (29/2,121) Europe, and 0.8% (12/1,506) North America (United States).

The MIC_90_ for sulbactam-durlobactam was 4 μg/mL for all antimicrobial-non-susceptible phenotypes of ABC studied, including sulbactam-non-susceptible (defined as MIC ≥ 8 μg/mL), carbapenem-non-susceptible, colistin-resistant, MDR, and XDR isolates (Table 2). At 4 μg/mL (the preliminary susceptibility breakpoint), sulbactam-durlobactam inhibited >96% of sulbactam-, imipenem-, ciprofloxacin-, amikacin-, and minocycline-non-susceptible, and colistin-resistant, MDR, and XDR isolates. Most imipenem-non-susceptible ABC isolates (96.8%, 2,488/2,570) were carbapenem-resistant A. baumannii (CRAB); 96.9% (2,410/2,488) of CRAB isolates were sulbactam-durlobactam-susceptible. At a concentration of ≤4 μg/mL, sulbactam alone inhibited <10% of isolates in all antimicrobial-non-susceptible phenotype subsets. The MIC_90_ value for sulbactam alone was 64 μg/mL for all ABC isolates tested and for all antimicrobial-non-susceptible phenotypes studied. Taken together, these results suggest there is little to no pre-existing cross-resistance between sulbactam-durlobactam and other classes of antimicrobial agents.

## DISCUSSION

The treatment of ABC infections is clinically challenging. Current first-line therapies include ampicillin-sulbactam, carbapenems (imipenem, meropenem), and broad-spectrum cephalosporins (ceftazidime, cefepime) when isolates demonstrate *in vitro* susceptibility (3, 21). In the collection of 5,032 ABC surveillance isolates tested in the current study, <50% were susceptible to sulbactam, cefepime, imipenem, meropenem, ciprofloxacin, and colistin (Table 1). Additionally, 53.3% (2,680/5,032) and 42.1% (2,116/5,032) of isolates, respectively, demonstrated MDR or XDR phenotypes (Table 2). Our results showing high rates of *in vitro* resistance to first-line therapies confirm those reported in earlier studies (4, 22) and reinforce the importance of identifying new therapies to treat ABC infections. Sulbactam-durlobactam has the potential to significantly lower the high incidence of difficult-to-treat resistance identified in ABC isolates (DTR; defined as intermediate/resistant *in vitro* to all β-lactam categories, including carbapenems, and fluoroquinolones) (23) by providing an active β-lactam-based therapy that would reduce reliance on less effective and more toxic reserve agents (aminoglycosides, colistin, tigecycline).

The current study reports *in vitro* susceptibility testing results for sulbactam-durlobactam against >5,000 clinical isolates of ABC collected in five global regions. Previously published reports have described the *in vitro* activity of sulbactam-durlobactam against far smaller, often regional isolate collections (<100 to 1,722 isolates) (11–16). The current study determined that a sulbactam-durlobactam concentration of 2 μg/mL (MIC_90_) inhibited 91.9% of 5,032 ABC isolates and that 98.3% of isolates tested with a sulbactam-durlobactam MIC value of ≤4 μg/mL, the preliminary susceptible MIC breakpoint for sulbactam-durlobactam (18, 19). The *in vitro* activity of sulbactam-durlobactam was shown to be consistent for A. baumannii and three additional ABC species, as well as for isolates across five geographical regions, isolates from five common infection sources, and isolates with multiple clinically relevant resistance phenotypes. Five of the six previously published studies also reported a sulbactam-durlobactam MIC_90_ of 1 to 2 μg/mL, irrespective of international clonal lineage, and the presence of various OXA-type β-lactamases in isolates, each study showing an MIC distribution similar to that shown in Fig. 1; a single study from Greece describing 190 carbapenem-resistant A. baumannii isolates reported an MIC_90_ of 8 μg/mL for sulbactam-durlobactam (15). The observation that durlobactam lowered the MIC of sulbactam for almost all (96.9%, 2,587/2,670) ABC isolates with sulbactam MICs of >4 μg/mL (sulbactam-non-susceptible) suggests that durlobactam inhibited class A, C, and D (OXA) β-lactamases in those isolates that would have otherwise hydrolyzed sulbactam (as well as imipenem, meropenem, and cefepime). In addition, these results collectively indicate that PBP mutations or MBLs in global isolates of ABC that can confer sulbactam-durlobactam resistance are currently rare, as discussed below.

In the current study, a small percentage (1.7%, 84/5,032) of isolates had sulbactam-durlobactam MIC values above the preliminary susceptible MIC breakpoint of 4 μg/mL (18, 19), an observation also reported in most earlier publications (11–14, 16). Clinical isolates with sulbactam-durlobactam MICs of >4 μg/mL have been shown to be comprised of two main types, and some may be clonal (12). The first group are isolates with mutations in PBP3 near its active site serine (S336) (24), the sulbactam-binding site. Common PBP3 mutations include A515V and T526S and confer sulbactam-durlobactam MICs of 8 to 32 μg/mL (12, 15). The second group are isolates that carry an MBL (NDM) and test with higher sulbactam-durlobactam MICs (32 to >64 μg/mL) (12). Currently, the prevalence of MBLs in ABC isolates is very low in most regions of the world (12, 25, 26).

In summary, the current study demonstrated the consistent and potent *in vitro* activity of sulbactam-durlobactam against recent, global clinical isolates of ABC. There are currently no reliably effective antimicrobial agents for the treatment of carbapenem-resistant A. baumannii infections. Our results suggest that sulbactam-durlobactam, if approved, may be useful for the treatment of infections caused by ABC, for which there is currently a high unmet medical need.

## MATERIALS AND METHODS

### Bacterial isolates.

From 2016 to 2021, 5,032 ABC isolates were collected by clinical laboratories in 264 medical centers in 33 countries (Table S4) and shipped to IHMA; 4,038 were A. baumannii, 638 were A. pittii, 296 were A. nosocomialis, and 55 were A. calcoaceticus. Four isolates of non-identified Acinetobacter species and one isolate of *A. dijkshoorniae* were also included in the collection. All isolates were cultured from patients receiving care in hospital and were limited to one isolate per infected patient. The identities of all isolates were confirmed by IHMA using matrix-assisted laser desorption ionization–time of flight mass spectrometry (Bruker Daltonics, Billerica, MA). Isolate collection employed annual, specimen source (bloodstream, intra-abdominal, lower respiratory, skin and soft tissue, and urinary tract), and geographic (country or region) quotas. Therefore, this study was not designed to evaluate the prevalence of individual species of Acinetobacter (or to estimate antimicrobial resistance) in the countries or regions from which participating laboratories supplied isolates to the study.

### Antimicrobial susceptibility testing.

The CLSI standard broth microdilution antimicrobial susceptibility testing method was used to determine MICs for sulbactam-durlobactam and nine comparator agents (27, 28). All testing was performed using cation-adjusted Mueller-Hinton broth in IHMA in-house-prepared custom broth microdilution panels (27, 28). Sulbactam-durlobactam was tested using 2-fold dilutions of sulbactam in combination with a fixed concentration of 4 μg/mL of durlobactam (27). MIC values for each agent were read and interpreted using CLSI standardized methods (27, 28). MIC breakpoint criteria are not currently published by CLSI for sulbactam-durlobactam, sulbactam alone, or tigecycline (27). Sulbactam-durlobactam MICs were interpreted using a preliminary susceptible MIC breakpoint of ≤4 μg/mL and a resistant MIC breakpoint of ≥8 μg/mL (18, 19). For comparative purposes, susceptible (≤4 μg/mL), intermediate (8 μg/mL), and resistant (≥16 μg/mL) MIC breakpoints were used for sulbactam (alone), based on the ampicillin-sulbactam (*in vitro* testing ratio 2:1)-susceptible, —intermediate, and -resistant breakpoints of 8/4, 16/8, and 32/16 μg/mL, respectively, where sulbactam is well established to comprise the active component of the combination against Acinetobacter spp. (27).

MDR isolates were defined as those not susceptible to agents from ≥3 different antimicrobial classes from the following list: cefepime (extended-spectrum cephalosporins), imipenem (carbapenems), amikacin (aminoglycosides), ciprofloxacin (fluoroquinolones), minocycline (tetracycline), sulbactam (penicillin plus β-lactamase inhibitor [sulbactam was used in lieu of ampicillin-sulbactam because it is the active component of the combination against Acinetobacter spp.]), and colistin (polymyxins) (29). XDR isolates were defined as those not susceptible to at least 5 of the 7 agents or agent classes listed above for MDR determination (i.e., isolates that were non-susceptible to ≥1 agent in all but ≤2 categories) (29). For colistin, only colistin-resistant isolates were used in MDR and XDR determinations because all isolates of Acinetobacter spp. are now classified as colistin-non-susceptible by CLSI (27).

### Statistical analysis.

The Cochran-Armitage test was used to assess linear trends in annual proportions of isolates with sulbactam-durlobactam MICs of ≤4 μg/mL from 2016 to 2021 (XLSTAT version 2020.2.1). A *P* value of <0.05 was considered statistically significant.
