# Supplementary material for: In Vitro Activity of Sulbactam-Durlobactam against Global Isolates of Acinetobacter baumannii-calcoaceticus Complex Collected from 2016 to 2021
Source: Antimicrob Agents Chemother. 2022 Aug 25;66(9):e00781-22. doi: 10.1128/aac.00781-22 (PMC9487466; doi:10.1128/aac.00781-22)
Supplement: Supplemental file 1 — Supplemental material. Download aac.00781-22-s0001.pdf, PDF file, 0.3 MB [file aac.00781-22-s0001.pdf]

SUPPLEMENTAL MATERIAL

TABLE S1 Regional analysis of *in vitro* activities of sulbactam-durlobactam and comparator antimicrobial agents tested against 5,032 clinical isolates of *Acinetobacter baumannii-calcoaceticus* complex (ABC) species collected globally from 2016 to 2021

Region (no. of isolates)	Antimicrobial agent	MIC (µg/ml)			MIC interpretation		
		MIC_{50}	MIC_{90}	Range	% Susceptible	% Intermediate	% Resistant
Asia/South Pacific (685)	Sulbactam-durlobactam[a]	1	2	≤0.03 – >64	98.4	NA[b]	1.6
	Sulbactam[c]	16	64	0.25 – >64	43.6	3.2	53.1
	Cefepime	>16	>16	0.25 – >16	42.6	2.3	55.0
	Imipenem	32	>64	0.06 – >64	43.9	0.4	55.6
	Meropenem	32	>64	0.06 – >64	44.2	0.3	55.5
	Amikacin	4	>64	≤0.5 – >64	55.8	0.7	43.5
	Ciprofloxacin	>4	>4	≤0.12 – >4	44.2	0.9	54.9
	Colistin[d]	0.5	1	≤0.25 – >8	NA	98.7	1.3
	Minocycline	0.5	8	≤0.12 – >16	86.7	8.6	4.7
	Tigecycline[e]	0.5	2	0.03 – 16	NA	NA	NA
Europe (2,121)	Sulbactam-durlobactam	1	4	≤0.03 – >64	98.6	NA	1.4
	Sulbactam	16	64	0.25 – >64	41.2	6.6	52.2
	Cefepime	>16	>16	≤0.12 – >16	40.6	7.0	52.4
	Imipenem	32	>64	≤0.03 – >64	43.9	0.5	55.5
	Meropenem	32	>64	≤0.03 – >64	43.4	0.8	55.8
	Amikacin	8	>64	≤0.5 – >64	51.6	1.8	46.6
	Ciprofloxacin	>4	>4	≤0.12 – >4	39.1	0.4	60.5
	Colistin	0.5	1	≤0.25 – >8	NA	93.3	6.7
	Minocycline	0.5	16	≤0.12 – >16	68.1	12.9	19.0
	Tigecycline	0.5	2	0.03 – 16	NA	NA	NA
Latin America (632)	Sulbactam-durlobactam	1	2	≤0.03 – >64	95.3	NA	4.7
	Sulbactam	16	64	1 – >64	28.8	11.4	59.8
	Cefepime	>16	>16	0.25 – >16	27.4	11.9	60.8
	Imipenem	64	>64	0.06 – >64	28.8	0.2	71.0
	Meropenem	64	>64	≤0.03 – >64	27.5	1.1	71.4
	Amikacin	32	>64	≤0.5 – >64	41.1	10.0	48.9
	Ciprofloxacin	>4	>4	≤0.12 – >4	27.4	0.8	71.8
	Colistin	0.5	1	≤0.25 – >8	NA	97.8	2.2
	Minocycline	0.5	8	≤0.12 – >16	83.5	7.6	8.9
	Tigecycline	0.5	2	0.03 – 8	NA	NA	NA

Region (no. of isolates)	Antimicrobial agent	MIC (µg/ml)			MIC interpretation		
		MIC_{50}	MIC_{90}	Range	% Susceptible	% Intermediate	% Resistant
Middle East (Israel) (88)	Sulbactam-durlobactam	1	2	0.25 – 32	97.7	NA	2.3
	Sulbactam	16	64	0.5 – 64	26.1	17.0	56.8
	Cefepime	>16	>16	1 – >16	23.9	9.1	67.0
	Imipenem	32	64	0.12 – >64	28.4	0	71.6
	Meropenem	64	>64	0.12 – >64	28.4	0	71.6
	Amikacin	32	>64	≤0.5 – >64	39.8	14.8	45.5
	Ciprofloxacin	>4	>4	≤0.12 – >4	23.9	0.0	76.1
	Colistin	0.5	1	≤0.25 – 2	NA	100	0
	Minocycline	1	8	≤0.12 – 16	89.8	4.5	5.7
	Tigecycline	0.5	2	0.06 – 2	NA	NA	NA
North America (1,506)	Sulbactam-durlobactam	1	2	≤0.03 – >64	99.2	NA	0.8
	Sulbactam	2	32	0.25 – >64	65.3	10.3	24.4
	Cefepime	4	>16	0.25 – >16	59.7	10.1	30.2
	Imipenem	0.25	64	0.06 – >64	67.9	0.9	31.3
	Meropenem	0.5	>64	0.06 – >64	65.7	1.9	32.4
	Amikacin	2	>64	≤0.5 – >64	78.2	3.3	18.6
	Ciprofloxacin	0.5	>4	≤0.12 – >4	60.4	1.0	38.6
	Colistin	0.5	1	≤0.25 – >8	NA	97.5	2.5
	Minocycline	0.25	8	≤0.12 – >16	86.0	8.2	5.8
	Tigecycline	0.25	2	0.03 – 32	NA	NA	NA

[a] Sulbactam-durlobactam MICs were interpreted using the preliminary MIC breakpoints of ≤4 µg/ml (susceptible) and ≥8 µg/ml (resistant).

[b] NA, not available.

[c] Sulbactam MICs were interpreted using the sulbactam component of CLSI M100 (2021) ampicillin-sulbactam MIC breakpoints (≤8/4 [susceptible], 16/8 [intermediate], and ≥32/16 [resistant]) given that sulbactam is well established to comprise the active component of the combination for *Acinetobacter* spp.

[d] CLSI M100 (2021) lists only intermediate and resistant MIC breakpoints for colistin tested against *Acinetobacter* spp.

[e] MIC interpretative criteria are not published by CLSI M100 (2021) for tigecycline tested against *Acinetobacter* spp.

TABLE S2 Specimen source analysis of *in vitro* activities of sulbactam-durlobactam and comparator antimicrobial agents tested against 5,032 clinical isolates of *Acinetobacter baumannii-calcoaceticus* complex (ABC) species collected globally from 2016 to 2021

Specimen source (no. of isolates)[a]	Antimicrobial agent	MIC (µg/ml)			MIC interpretation		
		MIC_{50}	MIC_{90}	Range	% Susceptible	% Intermediate	% Resistant
Bloodstream (1,015)	Sulbactam-durlobactam[b]	1	2	≤0.03 – >64	98.4	NA[c]	1.6
	Sulbactam[d]	8	64	0.25 – >64	49.5	7.4	43.2
	Cefepime	16	>16	0.25 – >16	48.2	6.1	45.7
	Imipenem	2	>64	0.06 – >64	50.9	0.6	48.5
	Meropenem	2	>64	0.06 – >64	51.0	0.4	48.6
	Amikacin	4	>64	≤0.5 – >64	60.6	3.3	36.2
	Ciprofloxacin	4	>4	≤0.12 – >4	49.2	0.7	50.1
	Colistin[e]	0.5	1	≤0.25 – >8	NA	95.8	4.2
	Minocycline	0.25	8	≤0.12 – >16	81.3	8.8	10.0
	Tigecycline[f]	0.5	2	0.03 – 16	NA	NA	NA
Respiratory tract (2,731)	Sulbactam-durlobactam	1	2	≤0.03 – >64	98.1	NA	1.9
	Sulbactam	16	64	0.25 – >64	41.6	8.3	50.1
	Cefepime	>16	>16	≤0.12 – >16	38.8	8.8	52.4
	Imipenem	32	>64	≤0.03 – >64	43.3	0.6	56.1
	Meropenem	32	>64	≤0.03 – >64	41.9	1.4	56.7
	Amikacin	8	>64	≤0.5 – >64	53.6	3.5	42.9
	Ciprofloxacin	>4	>4	≤0.12 – >4	38.8	0.6	60.6
	Colistin	0.5	1	≤0.25 – >8	NA	95.3	4.7
	Minocycline	0.5	16	≤0.12 – >16	75.4	11.5	13.1
	Tigecycline	0.5	2	0.03 – 32	NA	NA	NA
Urinary tract (832)	Sulbactam-durlobactam	1	2	≤0.03 – >64	98.9	NA	1.1
	Sulbactam	4	32	0.25 – >64	59.0	8.2	32.8
	Cefepime	8	>16	0.25 – >16	57.1	7.7	35.2
	Imipenem	0.5	64	0.06 – >64	62.7	0.6	36.7
	Meropenem	1	>64	0.06 – >64	61.7	1.0	37.4
	Amikacin	2	>64	≤0.5 – >64	71.0	3.5	25.5
	Ciprofloxacin	0.5	>4	≤0.12 – >4	54.9	1.2	43.9
	Colistin	0.5	1	≤0.25 – >8	NA	97.5	2.5
	Minocycline	0.25	16	≤0.12 – >16	82.7	7.2	10.1
	Tigecycline	0.25	2	0.03 – 16	NA	NA	NA

Specimen source (no. of isolates)[a]	Antimicrobial agent	MIC (µg/ml)			MIC interpretation		
		MIC_{50}	MIC_{90}	Range	% Susceptible	% Intermediate	% Resistant
Intra-abdominal (217)	Sulbactam-durlobactam	1	2	≤0.03 – 32	97.7	NA	2.3
	Sulbactam	16	64	0.5 – >64	41.0	7.8	51.2
	Cefepime	>16	>16	≤0.12 – >16	41.0	6.0	53.0
	Imipenem	32	>64	0.12 – >64	41.9	0	58.1
	Meropenem	64	>64	≤0.03 – >64	41.5	0.5	58.1
	Amikacin	8	>64	≤0.5 – >64	52.1	2.8	45.2
	Ciprofloxacin	>4	>4	≤0.12 – >4	39.2	0.5	60.4
	Colistin	0.5	1	≤0.25 – >8	NA	95.9	4.1
	Minocycline	0.5	8	≤0.12 – 16	79.7	13.4	6.9
	Tigecycline	0.5	2	0.03 – 4	NA	NA	NA
Skin and soft tissue (227)	Sulbactam-durlobactam	1	2	0.06 – 16	99.1	NA	0.9
	Sulbactam	4	64	0.5 – >64	60.8	6.6	32.6
	Cefepime	4	>16	0.25 – >16	55.5	8.4	36.1
	Imipenem	0.5	>64	0.12 – >64	63.0	0.4	36.6
	Meropenem	1	>64	0.06 – >64	61.2	1.8	37.0
	Amikacin	4	>64	≤0.5 – >64	69.2	1.8	29.1
	Ciprofloxacin	0.5	>4	≤0.12 – >4	57.7	0	42.3
	Colistin	≤0.25	1	≤0.25 – >8	NA	98.2	1.8
	Minocycline	≤0.12	16	≤0.12 – >16	81.9	6.2	11.9
	Tigecycline	0.25	2	0.03 – 8	NA	NA	NA

[a] 10 isolates were from specimen sources other than the five main specimen sources and their results do not appear in the table.
[b] Sulbactam-durlobactam MICs were interpreted using the preliminary MIC breakpoints of ≤4 µg/ml (susceptible) and ≥8 µg/ml (resistant).
[c] NA, not available.
[d] Sulbactam MICs were interpreted using the sulbactam component of CLSI M100 (2021) ampicillin-sulbactam MIC breakpoints (≤8/4 [susceptible], 16/8 [intermediate], and ≥32/16 [resistant]) given that sulbactam is well established to comprise the active component of the combination for *Acinetobacter* spp.
[e] CLSI M100 (2021) lists only intermediate and resistant MIC breakpoints for colistin tested against *Acinetobacter* spp.
[f] MIC interpretative criteria are not published by CLSI M100 (2021) for tigecycline tested against *Acinetobacter* spp.

TABLE S3 Annual antimicrobial susceptibility testing results for *Acinetobacter baumannii-calcoaceticus* complex (ABC) isolates collected worldwide from 2016 to 2021

Year	Antimicrobial agent	All isolates				*A. baumannii*				*A. calcoaceticus*				*A. nosocomialis*				*A. pittii*			
			MIC (µg/ml)				MIC (µg/ml)				MIC (µg/ml)				MIC (µg/ml)				MIC (µg/ml)		
		n	MIC_{50}	MIC_{90}	% MIC ≤4[a]	*n*	MIC_{50}	MIC_{90}	% MIC ≤4	*n*	MIC_{50}	MIC_{90}	% MIC ≤4	*n*	MIC_{50}	MIC_{90}	% MIC ≤4	*n*	MIC_{50}	MIC_{90}	% MIC ≤4
2016	Sulbactam-durlobactam	843	0.5	2	98.8	730	1	2	98.6	4	-[b]	-	100	22	0.25	0.5	100	87	0.5	1	100
	Sulbactam	843	8	32	42.0	730	16	64	29.4	4	-	-	100	22	1	4	95.5	87	2	4	93.1
2017	Sulbactam-durlobactam	826	1	4	97.0	637	1	4	96.1	6	-	-	100	38	0.5	1	100	145	0.5	2	100
	Sulbactam	826	8	64	48.9	637	16	64	37.4	6	-	-	100	38	2	16	73.9	145	2	4	91.0
2018	Sulbactam-durlobactam	929	1	2	99.3	697	1	2	99.4	13	0.5	1	100	54	0.5	1	100	164	0.5	1	98.2
	Sulbactam	929	4	64	50.8	697	16	64	36.9	13	2	4	100	54	2	8	85.2	164	2	4	94.5
2019	Sulbactam-durlobactam	859	1	4	97.8	699	1	4	97.4	13	0.5	1	100	68	0.5	2	98.5	80	0.5	2	100
	Sulbactam	859	8	32	44.5	699	16	64	34.8	13	4	4	92.3	68	2	16	77.9	80	2	4	93.8
2020	Sulbactam-durlobactam	795	1	2	98.2	638	1	2	98.0	12	0.5	1	100	66	0.5	1	100	76	1	2	98.7
	Sulbactam	795	8	64	47.8	638	16	64	38.2	12	2	4	91.7	66	2	8	83.3	76	2	8	89.5
2021	Sulbactam-durlobactam	780	1	2	98.8	637	1	2	98.6	7	-	-	100	48	0.5	1	100	86	0.5	1	100
	Sulbactam	780	8	64	47.4	637	16	64	38.0	7	-	-	85.7	48	2	16	81.3	86	2	4	95.3

[a]Percentage of isolates tested with an MIC ≤4 µg/ml.

[b] "-" indicates that MIC_{50} and MIC_{90} values were not calculated for numbers of isolates less than 10.

TABLE S4 Demographic information associated with 5,032 isolates of *Acinetobacter baumannii-calcoaceticus* complex (ABC) collected worldwide from 2016 to 2021

		No. of isolates [a]				
Parameter		All isolates	*A. baumannii*	*A. calcoaceticus*	*A. nosocomialis*	*A. pittii*
Specimen source						
	Bloodstream	1,015	757	13	85	160
	Intra-abdominal	217	180	2	6	28
	Respiratory tract	2,731	2,276	31	153	268
	Skin and soft tissue	227	174		14	39
	Urinary tract	832	644	9	38	140
	Other	10	7			3
	Total	5,032	4,038	55	296	638
Region	Country (number of participating medical centers)					
Asia/South Pacific	Australia (5)	97	59	3	13	22
	Japan (4)	71	45	2	16	8
	Malaysia (6)	85	76			8
	Philippines (4)	52	42		4	6
	South Korea (6)	135	126		6	3
	Taiwan (10)	113	87		14	12
	Thailand (4)	110	108		2	
	Vietnam (2)	22	22			
	Total	685	565	5	55	59
Europe	Belgium (7)	104	53	2	2	47
	Croatia (3)	20	20			
	Czech Republic (4)	116	92	1		23
	France (13)	243	157	1	6	77
	Germany (16)	218	125	8	4	81
	Greece (5)	150	149			1
	Hungary (6)	109	102		4	2
	Italy (14)	231	223		1	7
	Lithuania (2)	76	76			
	Poland (4)	85	80		4	1
	Portugal (5)	84	81		2	1
	Romania (4)	75	69			5
	Russia (13)	128	123		2	3
	Spain (19)	231	201	3	5	22

Parameter		No. of isolates [a] All isolates	*A. baumannii*	*A. calcoaceticus*	*A. nosocomialis*	*A. pittii*
	Turkey (7)	141	137		3	1
	Ukraine (3)	20	19			1
	United Kingdom (6)	90	56	4	3	27
	Total	2,121	1,763	19	91	
Latin America	Argentina (5)	119	110		3	6
	Brazil (13)	122	111		6	5
	Chile (3)	50	41		1	8
	Colombia (8)	84	68		14	2
	Guatemala (2)	120	117		2	1
	Mexico (7)	137	128	2		7
	Total	632	575	2	26	29
Middle East	Israel (5)	88	83		1	4
North America	United States (49)	1,506	1,052	29	178	247
	Total (264)	5,032	4,038	55	296	638
Year of isolation						
	2016	843	730	4	22	87
	2017	826	637	6	38	145
	2018	929	698	13	54	164
	2019	859	698	13	68	80
	2020	795	638	12	66	76
	2021	780	637	7	48	86
	Total	5,032	4,038	55	296	638

[a] There were 4 isolates of non-speciated *Acinetobacter* spp. and 1 isolate of *Acinetobacter dijkshoorniae* that are in the total data but not divided out in the table.
